# Migrated Laparoscopic Surgical Clips Causing Acute Cholangitis 32 Years After Laparoscopic Cholecystectomy

**DOI:** 10.7759/cureus.47415

**Published:** 2023-10-21

**Authors:** Yijie Yin, Su Kah Goh, Reizal Mohd Rosli, John-Edwin Thomson, Chuan Ping Tan

**Affiliations:** 1 Hepatopancreaticobiliary Surgery Unit, Royal Adelaide Hospital, Adelaide, AUS; 2 Department of Surgery, Austin Health, Melbourne, AUS

**Keywords:** acute pancreatitis, diagnostic and therapeutic ercp, acute cholangitis, endoclip, laparoscopic cholecystectomy (lc)

## Abstract

Surgical clip migration post-laparoscopic cholecystectomy is a rare but important complication to consider in patients presenting with biliary obstruction. Titanium surgical clips are widely used in laparoscopic surgery to ligate vessels and ducts and are particularly important in laparoscopic cholecystectomy to ligate the cystic duct. More common complications associated with clips involve dislodgement, however, there are reported cases of migration into visceral structures causing an obstruction. We describe a case that demonstrated an acute migration of surgical clips into the common bile duct (CBD) within a three-week period, which occurred 32 years after laparoscopic cholecystectomy, likely attributed to erosion. On the patient's first presentation, she had acute pancreatitis with a CT demonstrating clips in the correct position. Three weeks later, the patient presented a second time with acute cholangitis and the repeat CT demonstrated the clips in the CBD. We hypothesize that the erosion of the bile duct is due to the pressure effects from either intra-abdominal organ movements or subtle clip movements, and eventually, persistent erosion leading to intra-ductal migration of the clips with the passage of the clips along the path of least resistance into the CBD, resulting in biliary obstruction. Management included standard treatment for biliary obstruction with intravenous broad-spectrum antibiotics and endoscopic retrograde cholangiopancreatography with excellent outcomes.

## Introduction

Surgical clip migration after laparoscopic cholecystectomy is a rare complication. The first laparoscopic cholecystectomy was performed in 1985 by German surgeon Dr. Erich Mühe in Böblingen, Germany [1], and since then has become the gold standard for the treatment of gallstone-related disease. It is standard practice to use titanium surgical clips to ligate the cystic duct, with two to three clips placed onto the cystic duct stump to prevent bile leaks. Common surgical complications related to laparoscopic cholecystectomy include bile duct injury, bleeding, and bile leak. Rarely, the clips can migrate into the common bile duct (CBD), causing symptoms similar to choledocholithiasis and its associated complications. We describe a case that demonstrated an acute migration of surgical clips into the CBD within a three-week period, which occurred 32 years after laparoscopic cholecystectomy, likely attributed to erosion.

## Case presentation

**Figure 1:**
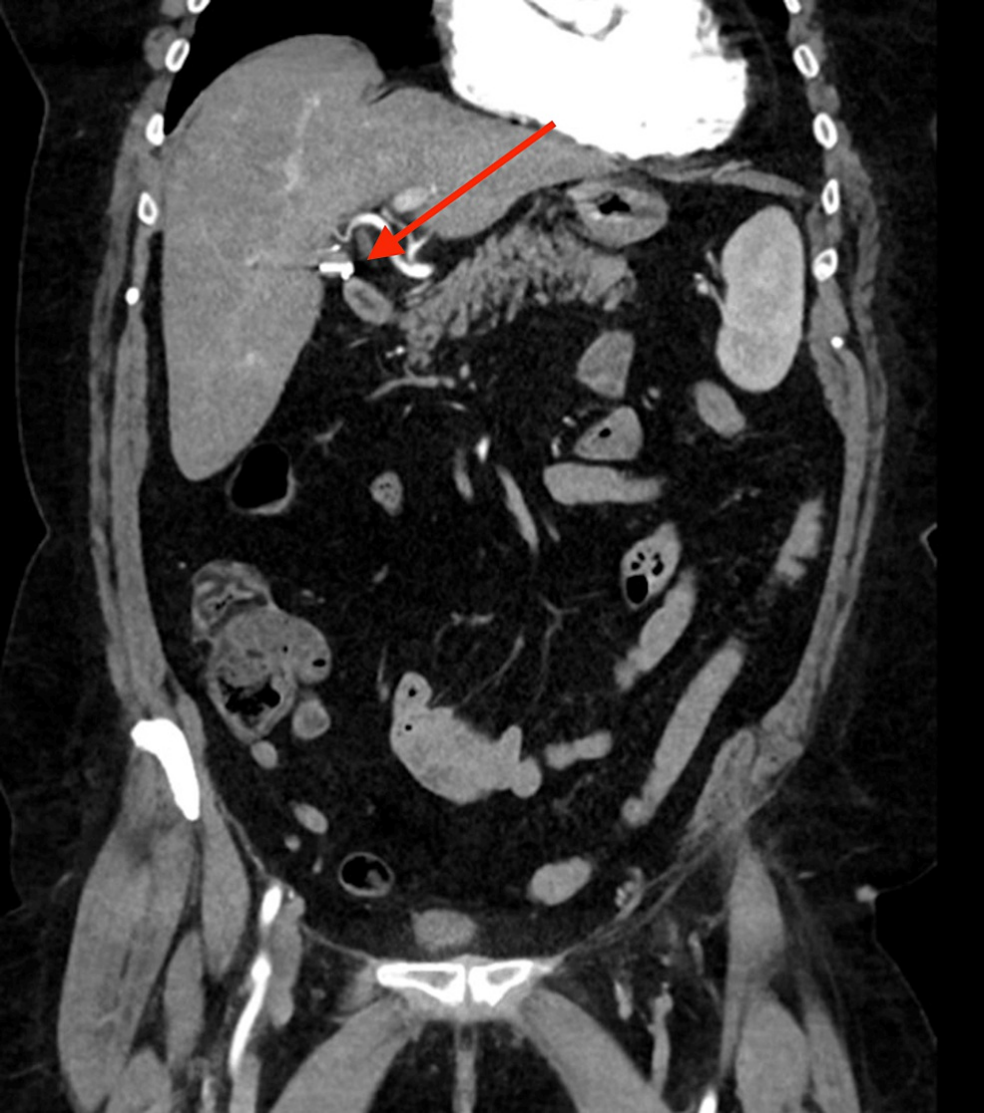
Computed tomography imaging of the patient’s first presentation demonstrating clips present at the cystic duct stump, as identified by the red arrow

A 70-year-old woman presented to a metropolitan tertiary hospital with acute pancreatitis. This was in the background of a laparoscopic cholecystectomy performed 32 years ago for cholecystitis. She was otherwise well, with no significant past medical history, and reported no alcohol intake. She presented with a two-day history of epigastric pain and vomiting. Biochemical results demonstrated obstructive liver enzymes, with a bilirubin of 151 µmol/L and serum lipase level greater than 1000 U/L. She was investigated with an ultrasound abdomen, computed tomography (CT) abdomen and pelvis, and magnetic resonance cholangiopancreatography (MRCP), all of which did not show choledocholithiasis or cholelithiasis, with a normal biliary system. Of note, the CT also confirmed the previous cholecystectomy with a surgical clip in the expected position (Figure 1). The patient was managed conservatively and discharged a day later following the resolution of her symptoms.

**Figure 2:**
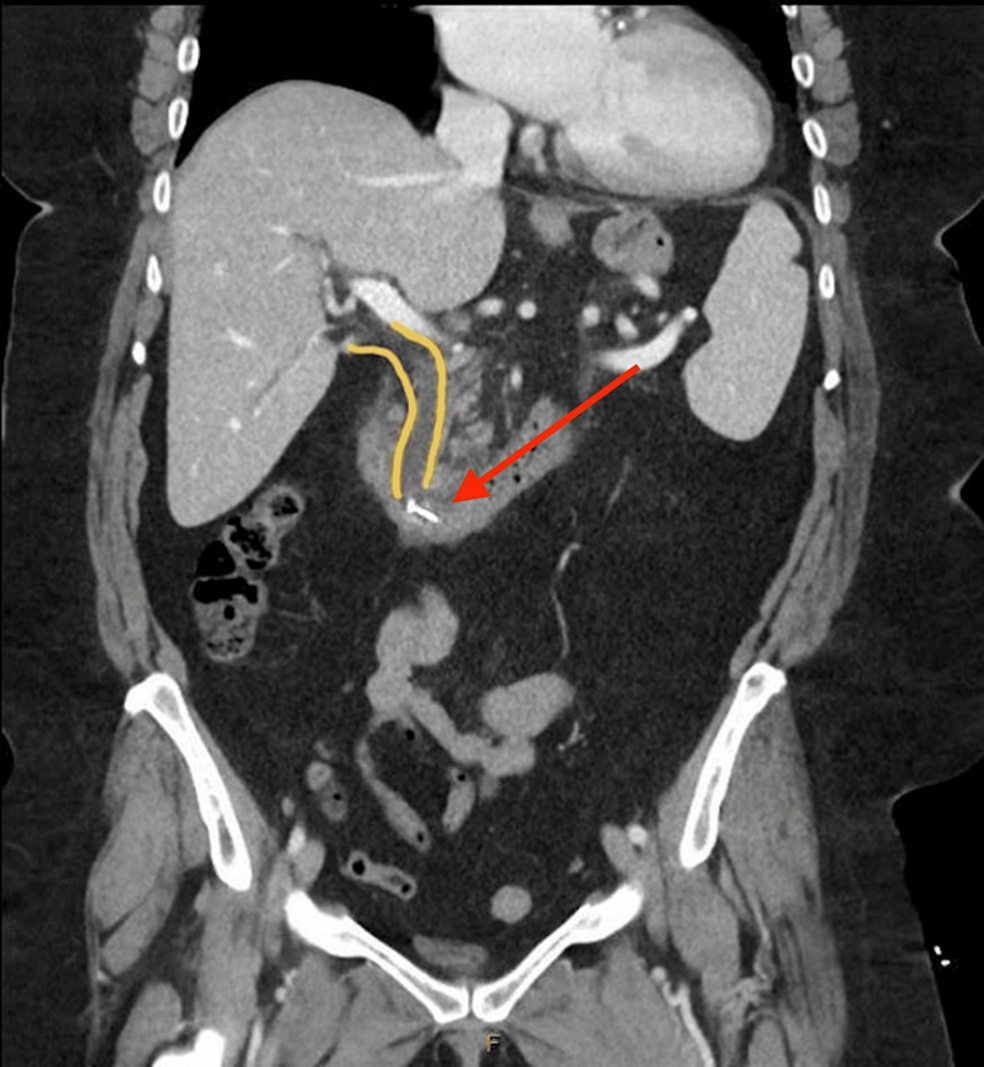
CT imaging of the patient's second presentation demonstrating a dilated common bile duct (CBD) as indicated by yellow lines with a migrated radio-opaque clip noted within the CBD, as shown by the red arrow

The patient re-presented three weeks later with Charcot's triad of right upper quadrant pain, pyrexia, and jaundice, suggesting acute cholangitis. Repeat biochemistry results again demonstrated obstructive liver enzymes (gamma-glutamyltransferase (GGT) 400, alkaline phosphatase (ALP) 260), hyperbilirubinemia (90 µmol/L)), and an elevated serum lipase level of 6500 U/L. She was also bacteremic. Blood cultures were positive for Streptococcus mutans. Another CT abdomen and pelvis was performed, and this demonstrated surgical clips that were placed during the cholecystectomy had displaced, eroded, and migrated into the distal CBD with resultant proximal extrahepatic and intrahepatic duct dilatation (Figure 2).

**Figure 3:**
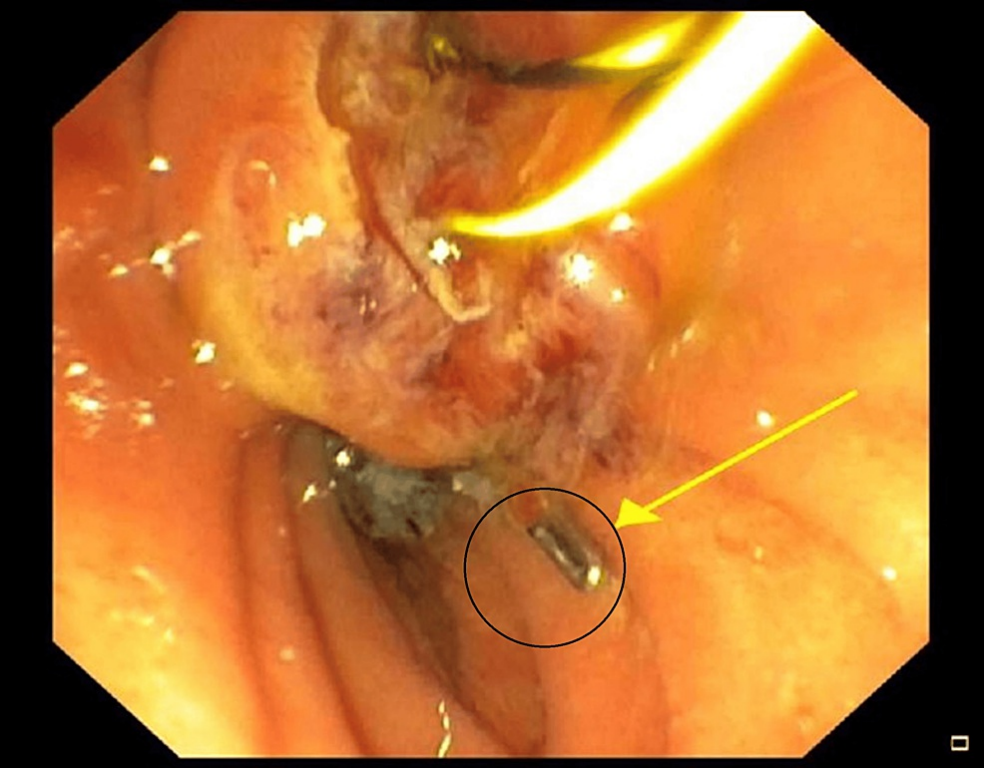
ERCP demonstrating a clip in the duodenum after being trawled from the CBD The migrated clip is demonstrated by the black circle and yellow arrow. ERCP: endoscopic retrograde cholangiopancreatography; CBD: common bile duct

Given the finding of migrated clips causing biliary obstruction and cholangitis, broad-spectrum antibiotics were commenced and an endoscopic retrograde cholangiopancreatography (ERCP) was performed. Purulent bile, two stones, and three clips were found within the CBD (Figure 3). A sphincterotomy was performed, and with balloon extraction, all the stones and surgical clips were trawled out. The patient had an unremarkable recovery and was discharged three days post-ERCP with full resolution of symptoms.

The patient was followed up in the outpatient clinic four months after her second admission. She had been able to return to her baseline function and activities of daily living, with no further reported episodes of abdominal pain.

## Discussion

Surgical clip migration after laparoscopic cholecystectomy was first reported in 1992 [2]. The literature suggests that clip migration can occur at any time, varying from 11 days to 20 years, with a median of two years [3]. The most common site of migration is into the CBD [4], where they can promote stone formation [3]. Other complications associated with clip migration include biliary colic, obstructive jaundice, cholangitis, and acute pancreatitis [4]. Clinical symptoms of clip migration are similar to the spectrum of gallstone pathology, with a majority of cases presenting with a combination of right upper abdominal pain, jaundice, and fever.

The causes of clip migration remain unclear. One hypothesis suggests that the erosion of the bile duct is due to the pressure effects from either intra-abdominal organ movements or subtle clip movement. Eventually, persistent erosion leads to intra-ductal migration of the clips with the passage of the clips along the path of least resistance [5]. Once in the CBD, the migrated clip acts as a nidus for stone formation, resulting in a biliary obstruction [5].

In this case, we postulate that the clip may have eroded over time, leading to the formation of stones within the cystic duct. The patient’s first presentation was potentially due to the passage of microlithiasis or radiologically translucent stones causing pancreatitis. The local inflammation and pressure effects may have resulted in an impetus for further clip erosion. The second presentation is attributable to the passage of the clips. Biliary obstruction and dislodgement of clips at the distal CBD would have caused pancreatic duct irritation, acute pancreatitis, and subsequent acute cholangitis. Based on the intervals of the scans, we can infer that the displacement of clips occurred within a three-week period.

Management of this case was based on the principles of management of acute pancreatitis and acute cholangitis. In the first presentation, as there was no radiological evidence of biliary obstruction and an improvement of biochemical findings of obstructive liver enzymes, it was appropriate to manage expectantly. The second presentation required more involved management of cholangitis and intervention with ERCP, as is routine with obstructive jaundice secondary to choledocholithiasis.

ERCP was useful in this case for clearance of the CBD and should be considered in the first instance. If initial attempts are not possible for clearance, a biliary stent should be considered for source control of sepsis. A repeat ERCP could be performed at a later stage for clearance of the CBD once oedema from pancreatitis has settled. Other options such as operative or percutaneous approaches should be reserved for settings where endoscopic approaches are not feasible or there are clinical contraindications against endoscopic approaches.

## Conclusions

This case highlights the need for consideration of the rare yet important differential of migrated surgical clips as the cause of biliary obstruction at any timeframe after laparoscopic cholecystectomy. It is suggested that retained foreign bodies, such as surgical clips, can erode and migrate through intra-abdominal tissues due to inflammatory reactions of tissues against the foreign body. Management is no different from any other routine presentation of biliary obstruction and cholangitis with an ERCP.
